# Effects of Prefabricated Versus Conventional Band and Loop Space Maintainers on Wound Healing, Plaque Accumulation, and Gingival Health of the Abutment Tooth: A Split-Mouth Randomized Controlled Trial

**DOI:** 10.7759/cureus.80308

**Published:** 2025-03-09

**Authors:** Kalyani Barve, Dimple Padawe, Vilas Takate

**Affiliations:** 1 Department of Pediatric and Preventive Dentistry, Government Dental College and Hospital, Mumbai, IND

**Keywords:** band and loop space maintainer, children, dental plaque, prefabricated space maintainers, space maintenance

## Abstract

Background: Conventional band and loop space maintainers are widely used for space maintenance but have drawbacks, such as soldering failures and multiple appointments. Prefabricated space maintainers address this by requiring less time and only one appointment. However, space maintainers may affect postextraction wound healing and plaque accumulation, leading to gingivitis.

Objective: This study compares the effects of prefabricated and conventional band and loop space maintainers on postextraction wound healing, plaque accumulation, and gingival health of the abutment tooth.

Methodology: The split-mouth study included 10 healthy children aged five to eight years with bilateral mandibular primary first molars indicated for extraction. Each patient's experimental side was randomly selected, dividing quadrants into two groups: conventional and prefabricated band and loop space maintainers. Follow-up appointments were scheduled on the third and seventh day after extraction to assess wound healing using the Landry index, and at first, second, and third months to assess plaque using the Plaque index and gingival health of abutment using the Gingival index. Statistical analysis was performed using Statistical Package for Social Sciences, version 21, for Windows (SPSS Inc., Chicago, IL). Overall intergroup comparison among two groups was done using unpaired t-test.

Results: Both prefabricated and conventional designs increased plaque accumulation and gingivitis. Conventional space maintainers resulted in more plaque accumulation and a higher incidence of gingivitis than the prefabricated ones. However, no statistically significant difference was observed in postextraction wound healing.

Conclusion: Prefabricated band and loop space maintainers offer a practical and efficient solution for space maintenance and represent a promising alternative to conventional designs.

## Introduction

The primary dentition is essential for a child's growth and development, influencing speech, mastication, appearance, and the proper eruption of permanent teeth [1]. The premature loss of primary teeth can lead to space loss and undesirable tooth movements, affecting occlusion. When early loss is unavoidable, space maintainers help preserve arch space and guide the eruption of permanent teeth, minimizing negative effects on occlusion [2].

The choice of space maintainer design depends on the patient’s specific needs [3]. Conventional band and loop space maintainers are widely used in pediatric dentistry for single-tooth loss due to their high success rate [4,5]. They offer several advantages, including their well-documented effectiveness in preserving space, preventing adjacent tooth drift, and maintaining proper occlusal development. Additionally, they provide good durability and stability when properly fabricated and cemented. However, they have drawbacks, including time-consuming fabrication, the need for multiple appointments, soldering failures, and the challenge of taking accurate impressions, especially in patients with a strong gag reflex [5].

To offset the disadvantages of conventional space maintainers, one of the latest innovations in banded space maintainers is prefabricated space maintainers. They require only one appointment, no laboratory work, and less time; are cost-effective; and provide hassle-free treatment, especially in uncooperative children [2]. However, prefabricated space maintainers also have certain limitations, such as limited adaptability to individual tooth morphology, potential issues with retention and fit, and the possibility of increased plaque accumulation due to their standardized design. Given these advantages and limitations, it is essential to evaluate their clinical performance, particularly in terms of wound healing, plaque accumulation, and gingival health, to determine their efficacy as a viable alternative to conventional space maintainers.

Whether it is a conventional or prefabricated space maintainer, these appliances lead to changes in the contours of the teeth, causing plaque accumulation and making it difficult to maintain oral hygiene, which can lead to dental caries and periodontal disease [6-8]. Since these space maintainers are placed immediately after extraction, they may affect postextraction wound healing as the presence of a foreign body is known to affect the wound healing process. The wound healing process occurs in distinct phases such as hemostasis, inflammation, proliferation, and remodeling. The wound healing process may be disrupted by continuous contact with space maintainers, potentially leading to delayed healing or localized inflammation. Furthermore, the presence of these appliances can contribute to periodontal complications, including gingival inflammation, hypertrophy, and recession, primarily due to plaque retention and mechanical irritation. Therefore, evaluating plaque accumulation using a standardized plaque index is crucial in assessing the oral hygiene challenges posed by space maintainers and their potential impact on periodontal health.

Despite the growing use of prefabricated space maintainers, limited evidence exists regarding their impact on these clinical parameters compared to conventional space maintainers. Hence, this study is planned to assess and compare the effect of prefabricated and conventional band and loop space maintainers on postextraction wound healing, plaque accumulation, and gingival health of the abutment tooth, providing valuable insights into their clinical efficacy and suitability for pediatric patients.

## Materials and methods

This study was a split-mouth randomized controlled trial conducted after approval from the Institutional Ethical Committee. It adhered to the ethical principles outlined in the Declaration of Helsinki and the guidelines of the Occupational Safety and Health Administration. Parents gave written informed consent before participating.

Sample size calculation

**Figure 1:**
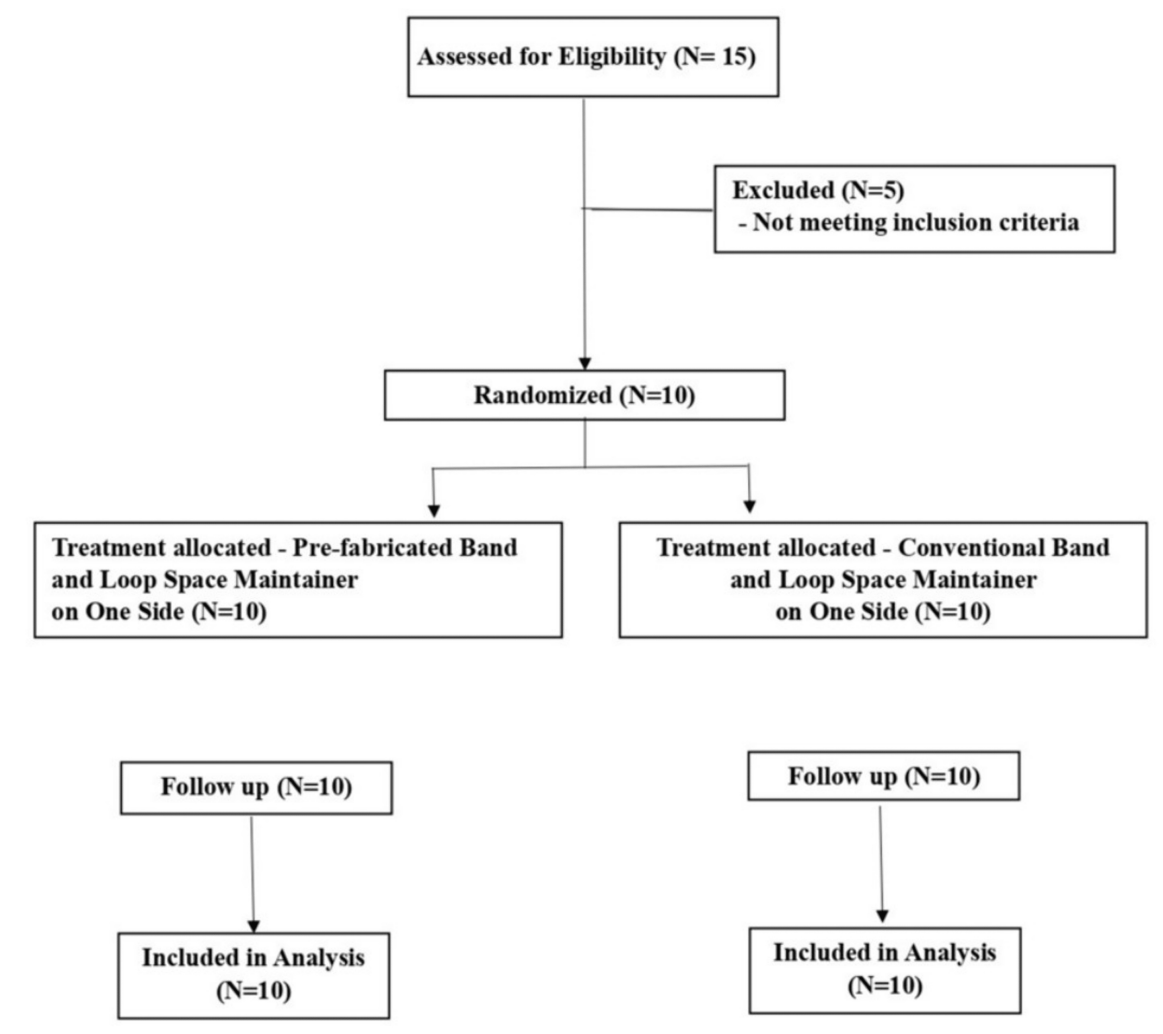
CONSORT flow diagram of the randomized split-mouth trial CONSORT: Consolidated Standards of Reporting Trials

A power analysis was conducted using G*Power version 3.0.1 (Franz Faul, Universität Kiel, Kiel, Germany). The total calculated sample size of 10 subjects (Group A: 10 sites, Group B: 10 sites) was determined to achieve 80% power for detecting significant differences, with an effect size of 1.35 and a significance level of 0.05. The effect size was derived from a similar split-mouth study by Tyagi et al., which compared conventional and modified space maintainers and reported comparable outcome measures (Figure 1) [9].

Study population and inclusion criteria

Children aged five to eight years with bilateral primary mandibular first molars indicated for extraction were included in the study, provided that a succedaneous tooth bud was confirmed radiographically, the abutment tooth was healthy, and adequate bone coverage was suggested a minimum of six months before permanent tooth eruption. Exclusion criteria included cases where the succedaneous tooth exhibited more than one-third root calcification, the space left by premature loss exceeded the space required for the permanent successor, the succedaneous tooth was absent, or the presence of medical conditions or special healthcare needs.

Study design and randomization

The study was conducted from June 1, 2024, to January 25, 2025. Ten healthy children meeting the eligibility criteria were enrolled. Before the intervention, each participant underwent a brief medical history assessment, clinical examination, and oral prophylaxis. A split-mouth design was employed, where each participant received both interventions in contralateral quadrants. Random allocation was performed using a computer-generated randomization sequence, and allocation concealment was ensured using sealed, opaque envelopes.

Intervention groups

**Figure 2:**
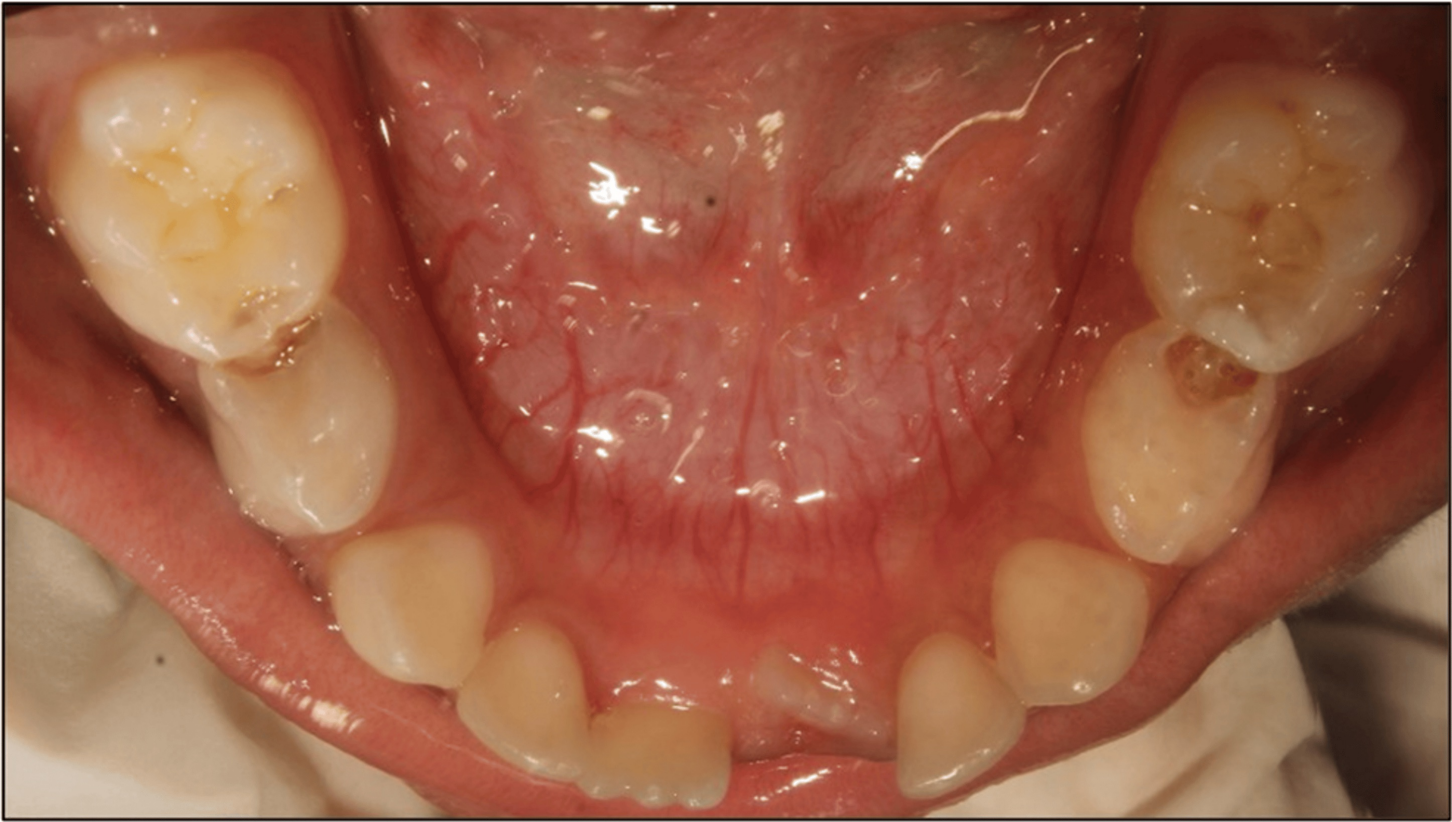
Preoperative photograph

Participants were randomly assigned to two groups (Figure 2): Group 1 (n = 10), who received a prefabricated band and loop space maintainer, and Group 2 (n = 10), who received a conventional band and loop space maintainer.

Group 1: Prefabricated Band and Loop Space Maintainer

Following the extraction of the mandibular primary first molar under local anesthesia, hemostasis was achieved. A preformed band was selected from the Kids-e Prefabricated Band and Loop Space Maintainer kit (Kids-e-Dental LLP, Mumbai, India) based on the mesiodistal width of the abutment tooth, measured using a Vernier caliper (Cometek, Diwakar Instruments Company, India). A prefabricated loop was selected according to the available mesiodistal and buccolingual space, inserted into the band’s tube, crimped, and cemented using GC Gold Label glass ionomer luting cement (GC Corporation, Tokyo, Japan).

Group 2: Conventional Band and Loop Space Maintainer

Fabrication was completed over two visits. In the first visit, a stainless-steel band was selected and adapted based on the mesiodistal width of the abutment tooth, measured using a Vernier caliper. An impression was made using alginate material, and a cast was prepared with the band stabilized using bobby pins [10]. The band and loop space maintainer was fabricated following Finn’s method, including soldering, finishing, and polishing of the appliance [11].

During the second visit, the extraction of the mandibular primary first molar was performed under local anesthesia, hemostasis was achieved, and the space maintainer was cemented using GC Gold Label glass ionomer luting cement.

Outcome assessment and follow-up

**Figure 3:**
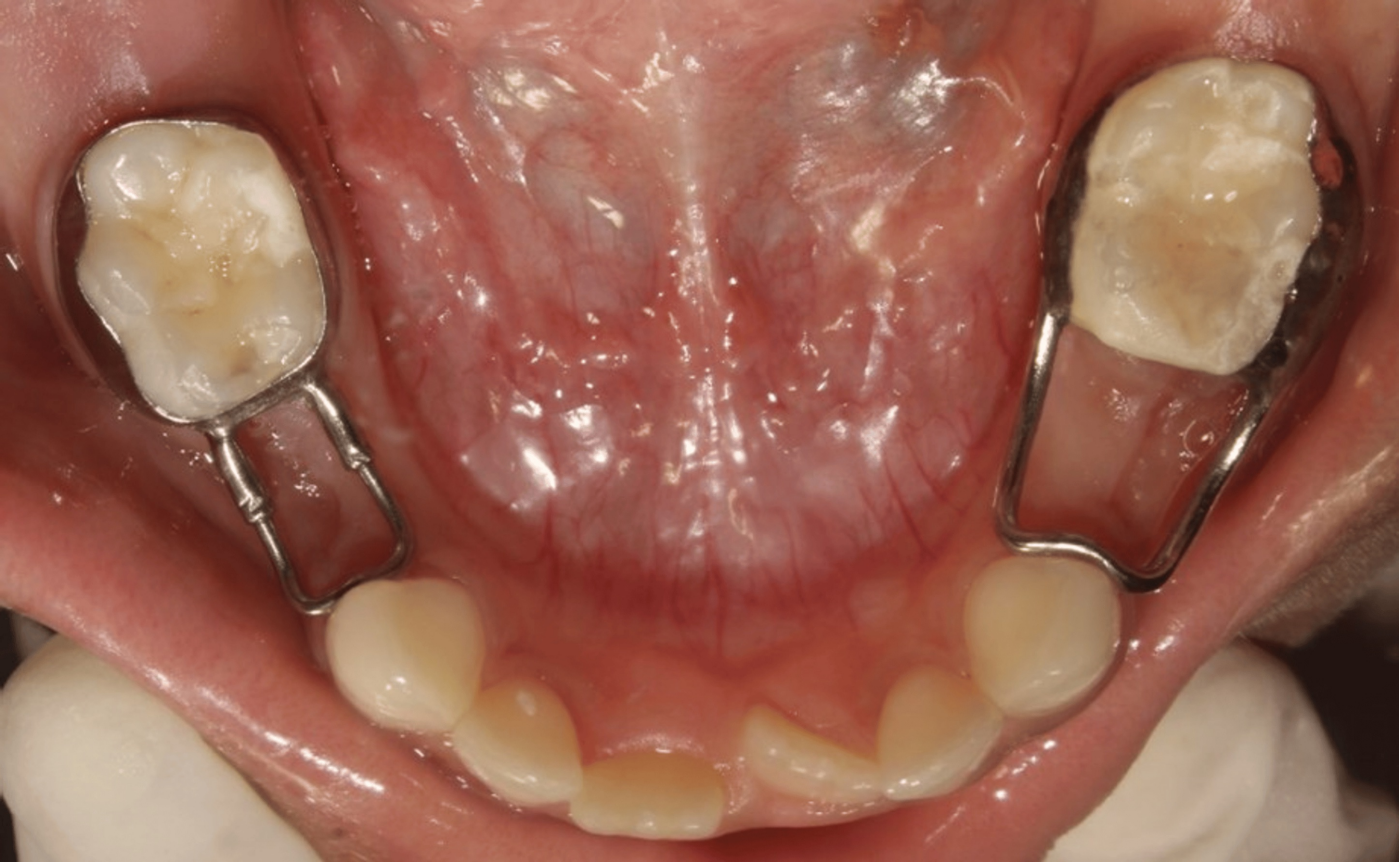
Postoperative photograph

Follow-up appointments were scheduled on the third and seventh days after extraction to assess wound healing and at the end of the first, second, and third months to evaluate plaque accumulation and gingival health of the abutment tooth in both groups (Figure 3). Wound healing was assessed using the Landry, Turnbull, and Hawley Healing Index (Table 1) [12].

**Table 1:** Wound healing index

Wound healing	Tissue color	Response to palpation	Granulation tissue
Very poor (score 1)	≥50% gingiva red	Bleeding	Present
Poor (score 2)	≥50% gingiva red	Bleeding	Present
Good (score 3)	≥25% and <50% gingiva red	No bleeding	None
Very good (score 4)	<25% gingiva red	No bleeding	None
Excellent (score 5)	All tissues pink	No bleeding	None

Plaque accumulation of abutment tooth was assessed using the Silness and Loe plaque index (Table 2) [13]. The score for the abutment tooth was calculated by adding the values from four areas of the tooth and dividing the total by 4. The interpretation of the scores was categorized as follows: a score of 0 indicated an excellent condition, scores ranging from 0.1 to 0.9 were considered good, scores between 1 and 1.9 were classified as fair, and scores from 2 to 3 were categorized as poor.

**Table 2:** Silness and Loe plaque index

Score	Criteria
0	No plaque
1	A film of plaque adhering to the free gingival margin and adjacent areas of the tooth. The plaque may be seen only by running a probe across the tooth surface
2	Moderate accumulation of soft deposits within the gingival pocket, on the gingival margin, and/or adjacent tooth surface, which can be seen by the naked eye
3	Abundance of soft matter within the gingival pocket and/or on the gingival margin and adjacent tooth surface

Gingival health was assessed using the Loe and Silness gingival index (Table 3) [13]. The score for the abutment tooth was determined by adding the values from four areas of the tooth and dividing the total by 4. The interpretation of the scores was as follows: a score between 0.1 and 1 indicated mild gingivitis, scores ranging from 1.1 to 2 indicated moderate gingivitis, and scores between 2.1 and 3 indicated severe gingivitis.

**Table 3:** Silness and Loe gingival index

Score	Criteria
0	Normal gingiva/absence of inflammation
1	Mild inflammation, slight change in color, slight edema, and no bleeding on probing
2	Moderate inflammation, moderate glazing, redness, edema and hypertrophy, and bleeding on probing
3	Severe inflammation, marked redness and hypertrophy, ulceration, and tendency to spontaneous bleeding

Statistical analysis

Statistical analysis was performed using Statistical Package for Social Sciences (SPSS) version 21 for Windows (SPSS Inc., Chicago, IL). Descriptive quantitative data were expressed in mean and standard deviation, respectively. Data normality was checked by using the Shapiro-Wilk test. The confidence interval is set at 95%, and the probability of alpha error (level of significance) is set at 5%. The power of the study was set at 80%. Overall intergroup comparison among two groups was done using unpaired t-test.

## Results

Healing index comparison

In the intragroup analysis, both Group 1 and Group 2 demonstrated a progressive improvement in healing from days 3 to 7, as evidenced by a significant increase in the healing index. The statistical analysis confirmed this, with a p value of 0.004 for both groups, indicating a statistically significant improvement in the healing process over time.

**Figure 4:**
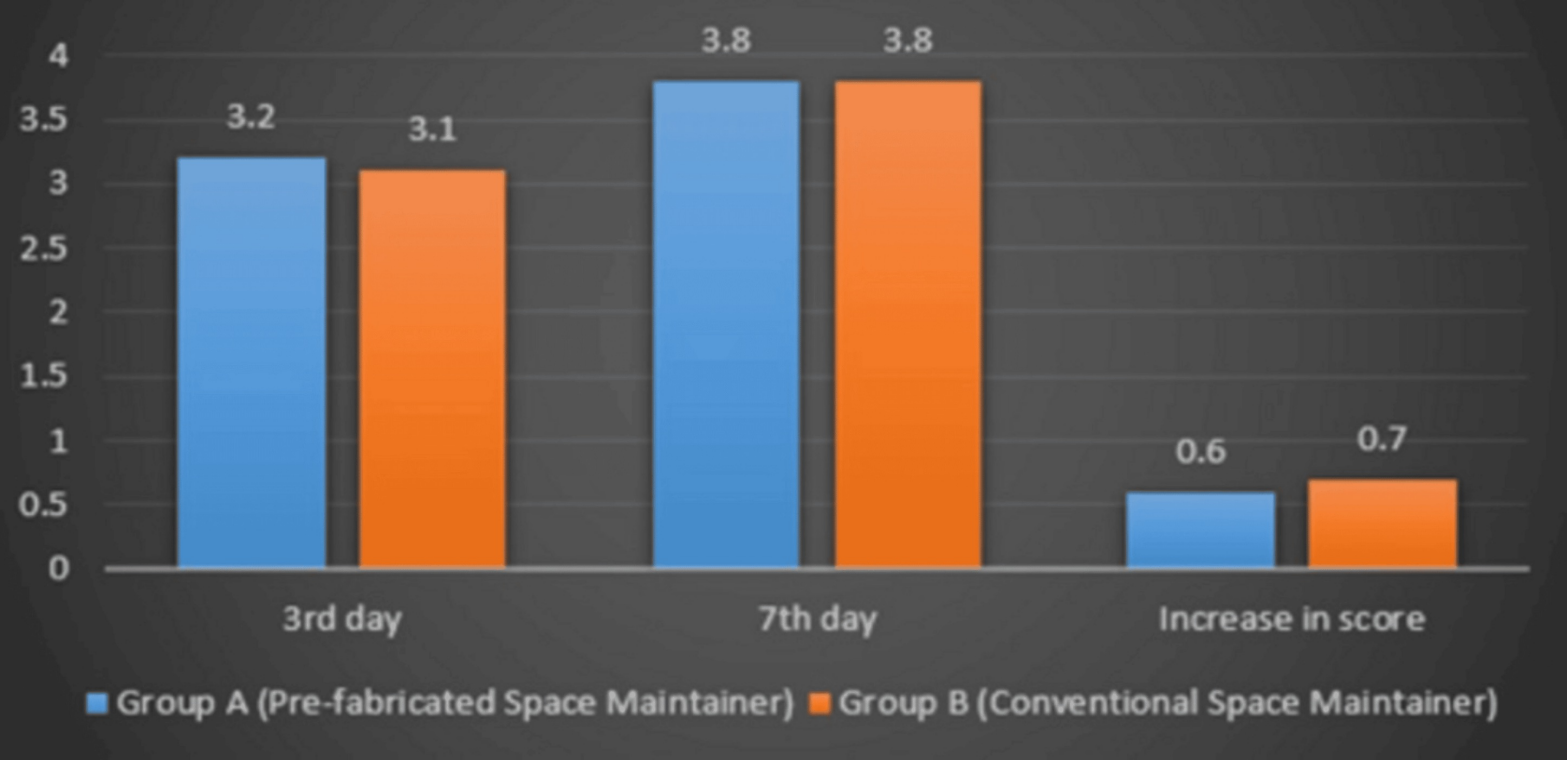
Healing index comparison

In contrast, the intergroup analysis revealed no statistically significant difference in the healing index between Group 1 and Group 2 at either time point. On days 3 and 7, the p value was 1.000, suggesting that both treatment modalities resulted in comparable healing outcomes. This indicates that neither intervention had a superior effect on wound healing within the observed timeframe (Figure 4).

Plaque index comparison

The intergroup analysis revealed a statistically significant difference in plaque accumulation between groups 1 and 2 at both the one- and three-month follow-up periods. At the one-month follow-up, Group 1 exhibited a significantly lower plaque index than Group 2 (p = 0.02), suggesting that the prefabricated band and loop resulted in less plaque accumulation during the early phase.

**Figure 5:**
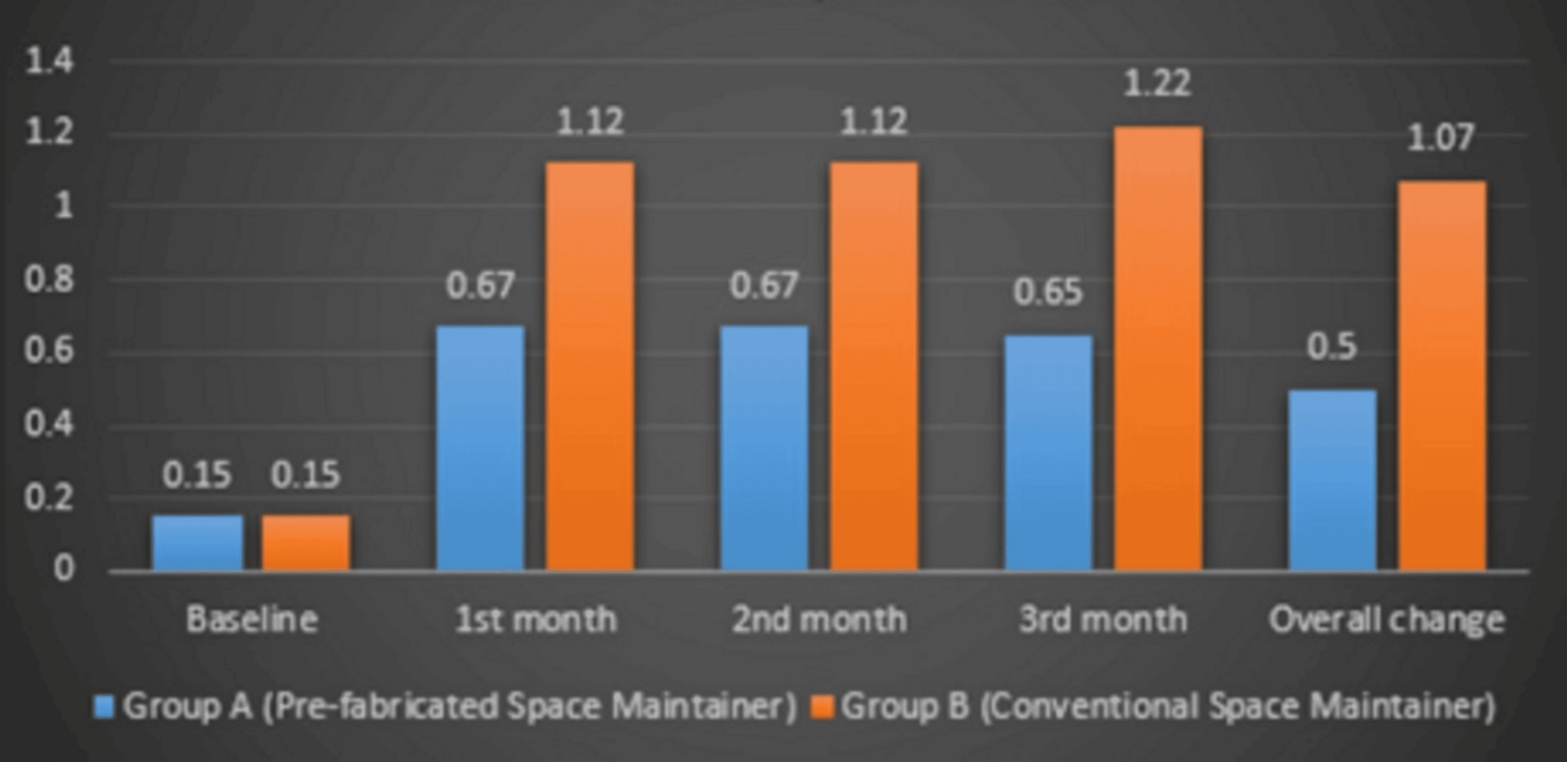
Plaque index comparison

This trend continued at the three-month follow-up, where Group 1 maintained a significantly lower plaque index than Group 2, with an even stronger statistical significance (p = 0.004). These findings indicate that the difference in plaque accumulation between the two groups persisted over time, potentially highlighting variations in material properties and surface smoothness (Figure 5).

Gingival index comparison

The intergroup analysis demonstrated a statistically significant difference in gingival health between Group 1 and Group 2 at both the one- and three-month follow-up intervals. At one month, Group 1 exhibited a significantly lower gingival index compared to Group 2 (p = 0.005), indicating better gingival health and reduced inflammation in this group.

**Figure 6:**
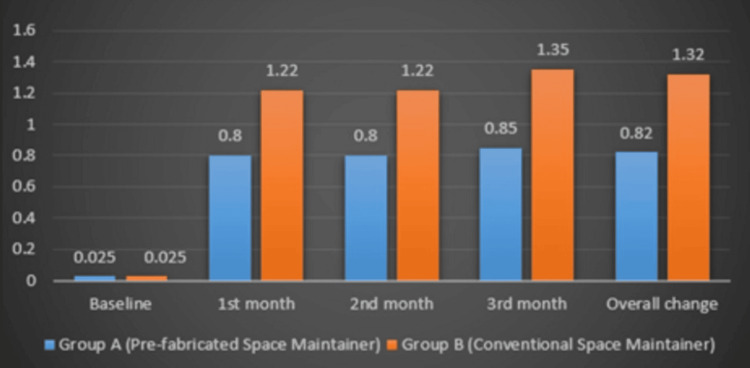
Gingival index comparison

This difference remained significant at the three-month follow-up, with Group 1 continuing to show a lower gingival index than Group 2 (p = 0.001). The sustained lower gingival index in Group 1 suggests that prefabricated band and loop may have better gingival outcomes, potentially due to factors such as reduced plaque accumulation, improved biocompatibility, or smoother material surfaces minimizing gingival irritation (Figure 6).

## Discussion

An ideal space maintainer should effectively preserve the mesiodistal space created following the loss of primary teeth while being structurally simple, durable enough to withstand functional forces, and supportive of normal occlusal function. Additionally, it should facilitate adequate oral hygiene, permit normal growth and adjustment of developing permanent teeth, and prevent supraeruption of opposing teeth or undue stress on adjacent teeth [3]. This study aimed to evaluate the effects of prefabricated and conventional band and loop space maintainers on postextraction wound healing, plaque accumulation, and gingival health.

Wound healing is a complex, highly regulated process driven by the body's natural repair mechanisms [14]. The findings of this study indicate no statistically significant difference in wound healing between the two groups. This similarity may be attributed to biocompatibility and the ability of stainless steel to integrate well with oral tissues, which is the primary material used in both prefabricated and conventional band and loop space maintainers [15]. Additionally, minimal tissue disruption caused by the placement of either appliance likely contributed to comparable healing patterns.

Further, comparing plaque accumulation and gingival health revealed that both prefabricated and conventional band and loop space maintainers led to increased plaque accumulation and gingivitis over follow-up periods of first, second, and third month. However, conventional band and loop space maintainers resulted in significantly higher plaque accumulation and greater incidence of gingivitis than the prefabricated ones. This finding is consistent with a previous study by Setia et al., where prefabricated bands and loops showed high levels of gingival health of 72.8% compared with conventional type of 45.7% within nine months [16]. A recent study by Dutta et al. revealed a significant statistical difference in gingival status between conventional and prefabricated band and loop space maintainers, wherein after a six-month follow-up, moderate gingivitis was observed in 8% of cases in the prefabricated group, compared to 12% in the conventional group [17].

Conventional band and loop space maintainers often exhibit an irregular surface due to the soldering process, which can contribute to increased plaque retention. The presence of soldered joints serves as a potential nidus for bacterial accumulation, thereby increasing the risk of gingivitis and periodontal disease [18-28]. Furthermore, inadequate polishing of soldered surfaces can result in additional surface irregularities that promote plaque accumulation [19-21,29,30]. In contrast, prefabricated band and loop space maintainers feature a smooth, uniform surface that enhances cleanability [16]. The absence of solder eliminates potential plaque-retentive areas, allowing for more effective plaque and debris removal, reducing the risk of gingival irritation and inflammation, and promoting better gingival health.

The findings of this study suggest that prefabricated band and loop space maintainers may offer advantages in reducing plaque accumulation and maintaining better gingival health compared to conventional band and loop space maintainers.

Strengths of the study

The strengths of this study include its split-mouth design, which minimizes interindividual variability and enhances the reliability of comparisons, as well as the use of objective assessment methods, such as standardized indices for plaque accumulation and gingival health. Additionally, the study holds significant clinical relevance, given the widespread use of space maintainers in pediatric dentistry. While evidence of their impact on soft tissues and wound healing remains limited, this study provides new insights into these aspects, emphasizing the need for further research to evaluate their long-term effects.

Limitations of the study

The limited sample size may restrict the generalizability of the findings to a wider population, as a larger cohort would improve statistical power and minimize the risk of sampling bias. Additionally, the short follow-up period may not adequately capture the long-term outcomes or delayed effects of the intervention. Future studies with a larger sample and extended follow-up are needed to validate these findings and assess the long-term effects of prefabricated and conventional band and loop space maintainers.

## Conclusions

Prefabricated band and loop space maintainers offer a practical and efficient solution for space maintenance, with distinct advantages in promoting better oral hygiene and gingival health. Their smooth, nonsoldered surface reduces plaque retention, lowering the risk of gingival inflammation.

In addition to clinical benefits, prefabricated space maintainers streamline treatment by eliminating the need for recording impressions and laboratory work, reducing chairside time, and enabling placement in a single appointment. This not only enhances workflow efficiency but also improves patient compliance and comfort. Given these advantages, prefabricated band and loop space maintainers represent a promising alternative to conventional designs, though further research is needed to evaluate their long-term effectiveness.
